# Effects of ink characteristics and piezo-electric inkjetting parameters on lysozyme activity

**DOI:** 10.1038/s41598-019-54723-9

**Published:** 2019-12-03

**Authors:** Tuser T. Biswas, Junchun Yu, Vincent A. Nierstrasz

**Affiliations:** Textile Materials Technology, Department of Textile Technology, Faculty of Textiles, Engineering and Business, University of Borås, Borås, Sweden

**Keywords:** Enzymes, Biomaterials - proteins, Enzymes, Biomaterials - proteins

## Abstract

Inkjet printing of enzymes can facilitate many novel applications where a small amount of materials need to be deposited in a precise and flexible manner. However, maintaining the satisfactory activity of inkjet printed enzyme is a challenging task due to the requirements of ink rheology and printhead parameters. Thus to find optimum inkjetting conditions we studied the effects of several ink formulation and jetting parameters on lysozyme activity using a piezoelectric printhead. Within linear activity range of protein concentrations ink containing 50 µg/mL lysozyme showed a satisfactory activity retention of 85%. An acceptable activity of jetted ink was found at pH 6.2 and ionic strength of 0.06 molar. Glycerol was found to be an effective viscosity modifier (10–15 mPa.s), humectant and protein structure stabilizer for the prepared ink. A non-ionic surfactant when used just below critical micelle concentration was found to be favourable for the jetted inks. An increase in activity retention was observed for inks jetted after 24 hours of room temperature incubation. However, no additional activity was seen for inkjetting above the room temperature. Findings of this study would be useful for formulating other protein-based inks and setting their inkjet printing parameters without highly compromising the functionality.

## Introduction

Controlled deposition of biological materials by inkjet printing has gained immense research interest in recent years^1,2^. This non-contact printing technology reduces contamination possibility of deposited biomaterials. Additionally, this digital printing technology enables minimum ink consumption, high-resolution graphical impression, and flexible production scale^3^. Accordingly, a range of biomaterials such as living cells, DNA/RNA, and proteins were recently explored for printing possibility^4,5^. A broad range of printed base-materials were explored for protein inkjetting e.g. polyethylene terephthalate films^6,7^, cellulosic papers^8^, and cotton fabric^9^. Most of this research was aimed at providing solutions to healthcare industry related applications such as microarray assay^10^, drug delivery^11^, and bio-sensors^12^. Among the bio-materials, inkjet printing of proteins, especially enzymes, have mostly been studied for their possible diverse applications in sensor fabrication, microarray patterning, combinatorial chemistry and biology, and drug formulations^1,2,13^.

During inkjet printing, enzymes need to withstand a variety of physio-chemical conditions inside printer machinery parts as an ink solution. Thus, printing parameters should be suitable for retaining the three-dimensional protein conformation to exhibit proper catalytic activity. Among two main classes of inkjet process i.e. continuous (CIJ) and drop-on-demand (DOD), the former one might cause unwanted electrostatic interaction with enzyme molecules^14^ as it relies on the conductivity of ink for drop formation. Additionally, DOD printers operate at lower frequencies and generate higher image resolution with smaller ink drops compared to CIJ systems^15^. DOD printers use a mechanical actuation technique for ink drop formation through a thermal or piezoelectric printhead. A thermal printhead may affect the three-dimensional protein structure of an enzyme due to high operating temperature and severe shear stresses generated in the ink liquid^16^. Comparatively, a piezoelectric system may influence such structural stability only due to the shear stresses^17^. These stresses may lead to higher fluid compression rate and damage the protein structure as found in the case of peroxidase by Nishioka^18^. Additionally, Arrabito *et al*.^19^ found activity loss for glucose oxidase (GOx) to be dependent on the voltage and waveform of such printhead. Conversely, Lonini *et al*.^10^ found no effect of shear stress on immunoglobulins (IgG) activity when compared between a single nozzle inkjet setup to manual pipetting. Thus, it is important to study the probable effect of shear stress during printing on activity for each enzyme.

Physio-chemical properties of the formulated ink define the printability and may become influential on enzyme activity. Theoretical printability of an ink can be understood through a series of limiting factors by calculating a group of dimensionless numbers e.g. Reynolds (Re), Weber (We), and inverse Ohnesorge number (Z), which have been widely discussed in literature^2,20–22^. Important is to realize that these calculated numbers depends on the ink viscosity, density, surface tension and characteristics length or print head orifice size. There is contradictory information in literature on defining the orifice size as nozzle diameter^1,20,23^ or radius^21,22,24^. However, a specific printing system would have a constant nozzle size and ink density of aqueous based enzyme ink would be dependent on the amount of viscosity modifier used. Therefore, viscosity and surface tension are the two most important properties of an ink solution to maintain an efficient printing process^15^.

Glycerol, polyethylene glycol, polyvinyl alcohol, and sodium carboxymethyl cellulose (CMC) are common viscosity modifiers for ink formulation. Di Risio and Yan^25^ found that activity of horseradish peroxide (HRP) was significantly reduced with increased amounts of these viscosity modifiers, though only one ink solution containing a low amount of CMC was printed in the study. Arrabito *et al*.^19^ found a similar trend of activity reduction for printing GOx with a higher proportion of glycerol. To maintain proper surface tension, non-ionic surfactants are preferred for enzyme-based inks as they would cause less unwanted interaction with the protein conformation^14^. The concentration of surfactant used in the ink solution could also be a critical factor to influence enzymatic activity^26^. However, to our knowledge, there are no studies concerning the factor of surface tension on the activity of inkjetted enzyme. Along with the rheological factors, the ionic nature of the ink can influence the activity of the printed enzyme. Ionic strength (*I*) and pH of ink solution may regulate movements of charged molecules along the active site of enzyme and substrate and thereby, adsorption, activity mechanism and stability of the three-dimensional protein conformation^27,28^. In addition to these physio-chemical properties, ink storage and print head operating conditions may affect activity. Though most of the enzymes maintain the best activity when stored at low-temperature conditions^29^, an inkjet printing process is preferred at temperatures higher than room temperature^15^. Therefore, it is important to study the effects of these parameters on printed enzyme activity.

In this paper, we present a comprehensive study on the activity retention of lysozyme subjected to a range of ink and jetting process parameters i.e. protein concentration, pH-*I* profile, rheology, storage condition, and printhead temperature. Lysozyme was selected as a model enzyme as its physio-chemical natures are well-studied^27,30,31^. Though its behaviour upon piezoelectric inkjet printing has not been investigated prior to this study. Our findings show that along with jetting stress, activity of lysozyme is affected by several of the above mentioned parameters.

## Experimental

### Materials

Lysozyme from chicken egg white (E.C. 3.2.1.17) and *Micrococcus lysodeikticus* cell (MLC) for enzyme activity assays were purchased from Alfa-Aesar (Germany) and Sigma-Aldrich (Germany) respectively. Protein quantification was conducted using a Bicinchoninic acid assay kit (BCA) manufactured by BioVision, Inc. (USA). Glycerol (≥99.0%), and polyethylene glycol (PEG, Mw 400, Reagent grade) was used as viscosity modifiers and Triton™ X-100 (Reagent grade) was used as the surfactant. Potassium dihydrogen phosphate (for pH 5–8), sodium carbonate (for pH > 8), hydrochloric acid and sodium hydroxide were used to prepare buffer solutions. All were purchased from Sigma-Aldrich (Germany). Milli-Q water (18–20 MΩ.cm at 25 °C) was used to prepare buffer solutions and for cleaning.

### Methods

#### Ink preparation and characterization

Ink for enzyme was prepared by adding an adequate amount of buffer solution (pH 6.2, ionic strength 0.05), viscosity modifier, and surfactant to maintain the viscosity of 12 mPa.s and surface tension of 32 mN/m (if not mentioned otherwise). Ink pH was measured using a Mettler Toledo F20 (USA) pH meter. Ionic strength was calculated as a concentration function of all the ions present in ink solution. Viscosity and surface tension were measured using a modular compact rheometer (Physica MCR, Anton Paar GmbH, Austria), and an optical tensiometer (Attension Theta, Biolin Scientific, Sweden), respectively. Viscosity measurements were conducted at 25 °C under a constant shear rate of 10000 s^−1^. The pendant drop method with an ink drop volume of 4 μL was used to measure surface tension. Then, lysozyme dissolved in buffer solution was added to the ink vehicle, resulting in a protein concentration of 50 µg/mL (if not mentioned otherwise). All measurements were done in triplicates.

#### Inkjet printing

A printer platform manufactured by Xennia technology was used. The platform enables three directional adjustments of printhead carriage and allows printing on a base-material as thick as five centimetres. Inks were supplied into printhead directly from glass bottles through inert plastic tubing. For printing, we used a Dimatix Sapphire QS-256/80 AAA (Fujifilm, USA) piezoelectric printhead with 80 pL native drop size and 100 dpi resolution. Thorough cleaning of printhead and tubing were done when switching between samples. Ink solution of 30 mL was purged to remove the traces of previous ink, followed by purging 20 mL of the ink to be jetted. Jetting was performed at 25 °C (if not mentioned otherwise) and after multiple printhead passes inks were collected on a glass plate as a rectangle shaped solid pattern. All samples were printed in duplicates. Jetting voltage and waveform were constant for all samples.

#### Protein concentration assay

The protein concentration of inkjetted samples was counted by using BCA assay technique^32^ to correct for any variation due to evaporation effect during jetting. A working solution was made by adding 50 parts of reagent A (sodium carbonate, sodium bicarbonate, bicinchoninic acid and sodium tartrate in 0.1 M sodium hydroxide) and 1 part of reagent B (cupric sulfate). Ink sample of 0.1 mL was added to 2.0 mL of working solution and incubated at 37 °C for 30 minutes before cooling to room temperature. The concentration of protein was measured by the corresponding absorbance at 562 nm against a constructed standard curve. The results were then normalized to respective non-jetted inks for calculation of lysozyme activity.

#### Enzyme assay

MLC substrate solution of 0.01% (w/v) was prepared with 66 mM phosphate buffer pH 6.2. In a cuvette (1 cm light path), 0.10 mL of ink solution was added to 2.5 mL of substrate solution and mixed well by inversion. Lytic activity of lysozyme inks was recorded as a decrease in absorbance at 450 nm for 4 minutes (one-second interval) by a UV–Vis spectrophotometer (Evolution 201, Thermo Scientific, USA). During recording, duration cuvettes were equilibrated at 25 °C using a Peltier controller unit (Evolution, Thermo Scientific, USA). One active unit was defined as the amount of enzyme causing a decrease in absorbance of 0.001 per minut. Activity units were calculated from the initial linear rate against a standard calibration curve covering protein concentration between 5 to 300 µg/mL.

#### Data analysis

The OriginLab program was used for data analysis. All presented data points are the mean of three observations (n), and an error bar represents standard deviation. Percent relative standard deviation was used for activity (%) data sets as (100* σ)/$\bar{{\rm{x}}}$ and Gauss approximation (propagation uncertainty) for reduction (%) data sets as Eq. 1.

1 $$\sqrt{Var(\frac{\bar{y}}{\bar{x}})}\approx \sqrt{{(\frac{{\mu }_{y}}{{\mu }_{x}^{2}})}^{2}\cdot \frac{{\sigma }_{x}^{2}}{{n}_{x}}+{(\frac{1}{{\mu }_{x}})}^{2}\cdot \frac{{\sigma }_{y}^{2}}{{n}_{y}}}$$

Here, x and y are independent; σ is standard deviation the of sample mean; µ is standard uncertainty.

Comparisons between two groups were done by Student’s t-test and one-way ANOVA for multiple comparisons. Tukey’s analysis was used to determine the level of significance and values of p < 0.05 regarded as significantly different.

## Results and Discussions

### Ink printability

Theoretical printability of all the prepared ink were calculated by Eqs. 2–5 as suggested in literature^20–22^.

2 $$Re=\frac{v\rho r}{\eta }$$

3 $$We=\frac{{v}^{2}\rho r}{\gamma }$$

4 $$Z=\frac{Re}{\sqrt{We}}$$

5 $$K=W{e}^{1/2}\,R{e}^{1/4}$$

Here, Re, We, Z and K are Reynolds number, Webers number, inverse Ohnesorge number and splashing parameter respectively. Velocity, density, characteristics length, and surface tension are denoted by η, ρ, r and γ respectively. Print head nozzle radius was used as characteristics length in our calculations as it was used in the earliest literature^21^ on this topic and accordingly established in several experimental works^22,24^.

Efficient printability of a DOD ink depends on four major limiting factors as calculated in Table 1. Fromm^21^ was the first to suggest that *Z* > 2 was required for stable drop formation and later extended to an acceptable range of 1 < *Z* < 10 by Derby^24^ by numerical simulation. Value of *Z* < 1 would prevent drop ejection due to viscous dissipation and *Z* > 10 would result in satellite drops. However, there are recent studies that suggest that *Z*≫ 10 may permit effective drop generation on significantly different ink forms and printing devices.^22,23^. Next, a drop must overcome the influence between air/fluid interface and associated Laplace pressure for proper ejection that can be defined by *We* > 4 as proposed by Duineveld^33^. Finally, impact of the drop on printed base material should be considered to avoid splashing effect on the graphic. Thus, it is highly dependent on base material surface roughness and require thorough research to find a onset of splashing. To avoid such splashing an experimental threshold of *K* < 50 has been suggested by Stow^34^ for flat and smooth surfaces. These limiting factors results in a printable window of the discussed parameters. As presented in Table 1, all our prepared inks were well within this permitted window and hence indicated efficient printability.

**Table 1:** Calculated printability values of the prepared inks from Eqs. 2–5.

*Z*	*Re*	*We*	*K*
1.1–3.7	3.7–12.9	6.8–15.3	2.0–3.3

### Protein concentration and linear activity range

Initial velocity, reversibility, and substrate saturation of enzymatic reaction depends on the enzyme concentration in the system and thus defining a linear range of activity detection^35^. Hence, it is necessary to determine a concentration that will ensure a constant reaction rate and maximum possible activity after printing. Accordingly, several lysozyme inks of varied protein concentration (5–100 µg/mL) were jetted in this study. All the concentrations were readily soluble and appeared transparent in the formulations of ink-making solvents, which provided assurance against nozzle clogging issues. Moreover, the particle size of this enzyme is compatible with nozzle diameter of used printhead to reduce the probability of such issues^36,37^.

Another concern for water-based enzymatic inks is that they are susceptible to show higher protein number afterwards due to evaporation of water molecule during printing. We experienced such effect initially that showed higher activity values after jetting even when humectants were used in the formulation. It is not clear in the protein inkjet printing literature if this evaporation effect was included in activity calculations. Thus, to exclude such discrepancies, we measured the protein concentration of the printed ink by a separate assay to normalize all activity calculations.

**Figure 1:**
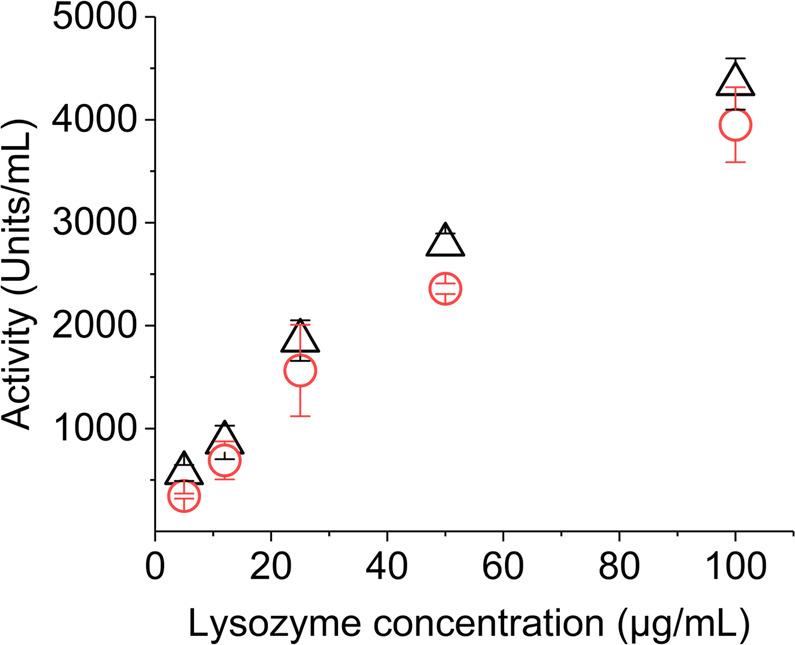
Lytic activity of lysozyme ink before (∆) and after (○) inkjetting at 25 °C for protein concentrations of 5–100 µg/mL. Error bars represent standard deviation.

The results showed an expected linear increase of activity until protein concentration of 50 µg/mL both for freshly prepared and jetted inks (Fig. 1). However, all the inks experienced a significant reduction in activity after jetting. The reduction was almost 40% for inks below 12 µg/mL of lysozyme and then gradually improved with increased concentration. Optimum concentration was found to be 50 µg/mL with the highest remaining activity of about 85% within the linear range. In general, inks containing 12–50 µg/mL lysozyme can be regarded as feasible for jetting through this printhead with the scope of further activity improvement by optimizing other ink properties and jetting parameters.

Activity reduction among the inks in Fig. 1, could be caused by the shear stress during jetting process and/or surface adsorption of lysozyme molecules inside the piezoelectric printhead. Though there is an indication in the literature about protein adsorption inside printhead surface^38^, it was less like to occur in our experiments as all the tubing and printhead inner surfaces were well purged with the ink prior to jetting (see method section). Additionally, the probability of such variations was normalized through protein concentration assay as already discussed. Alternatively, shear stress caused by the jetting force of piezoelectric element for drop generation might have been more influential for alteration of lysozyme protein structure and related activity reduction^39^. The reduction effects were much severe for inks with lower protein concentrations. Though the intensity of shear stress was analogous to all inks, probably it was more evident for inks with lower protein concentrations. Therefore, to ensure the best activity retention, the highest protein concentration within the linear activity range could be selected for inkjetting.

A general comparison of the probable effect of piezoelectric and thermal inkjet printing on lysozyme activity can be made from the findings of Fig. 1. Piezoelectric printhead used in our study maintained at least ten times higher lysozyme activity than its thermal counterpart (ca. 7% activity for 50 µg/mL) as found by Montenegro-Nicolini *et al*.^40^. It indicates that the protein structure of lysozyme might be more stable to shear stress inside a piezoelectric printhead, compared to the high heat conditions inside a thermal printhead.

### Ink pH and ionic strength optimization for improved activity

Amphoteric nature of the enzyme surface can vary depending on their acid dissociation constant (pK_a_). Thus, the pH of ink solution can influence the amount of net surface charge around amino acid residues of the enzyme according to its pK_a_ value. Therefore such pH change may affect solubility, structural stability and most importantly their catalytic activity of an enzyme^28^. To know about the probability of such effect, six lysozyme inks were jetted at evenly spaced pH interval (5.2–10.2) with optimized protein concentration.

**Figure 2:**
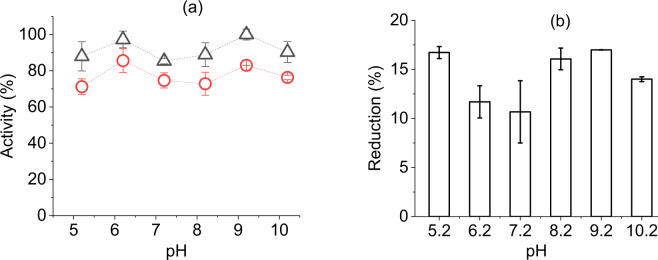
Effect of pH on the activity of inkjetted lysozyme, (**a**) relative activity before (∆) and after (O) jetting when expressed as a percentage of the highest value observed, (**b**) activity reduction after jetting process. Error bars represent standard deviation.

The two most significant ink pH values which showed relatively higher activity values both before and after jetting units were 6.2 and 9.2 (*I* = 0.05 M) as shown in Fig. 2. This is in agreement with the behaviour of lysozyme in buffer solution^30,31,41^. It has been explained in previous studies^27,30^ that lytic activity of oppositely charged lysozyme and its substrate depends on electrostatic forces acting between their surfaces to facilitate initial adsorption. Accordingly, an increase of pH value caused lysozyme to reach near its isoelectric point (pH~11) and thereby reduced adsorption probability to exhibit activity, except at pH 9.2. Along with pH, the salt concentration of the ink solution can regulate the necessary electrostatic interactions to impact activity units. The ionic strength (*I*) used for the inks of Fig. 2 was most electrostatically favorable at pH 9.2^30^ and hence showed relatively higher activity number than other pH levels. However, at this pH, activity reduction percentage after jetting were significantly higher compared to pH 6.2 (Fig. 2), indicating probable differences on the vulnerability of lysozyme protein structure to jetting force at varying pH levels. An explanation for such a phenomenon can be offered in term of protein stability, defined as the difference in Gibbs free energy between folded and unfolded protein structure^42^. The proton donating residue group has been identified to be dissimilar around these two pH ranges^43^. As discussed by Yang and Honig^44^ and Goyal *et al*.^45^, a variation of pH can significantly influence the net change on ionisable residues to distress the unfolding free energy. Their studies have demonstrated that for lysozyme the net charge on residues and difference in free energy can be varied around pH 6 and 9. Titration curve results in their studies also indicated that a pH beyond pK_a_ values of side chains may cause the residues to be buried and thus, destabilizing the protein structure or vice-versa. In another study, the transition temperature of lysozyme unfolding decreased around pH 9, affecting stability^46^.

The results in Fig. 2 indicate that although lysozyme activity might be slightly higher at pH 9.2 than at pH 6.2, structural stability of the enzyme might have been inferior at pH 9.2 ink to withstand the jetting force. The increase of activity under certain conditions at the expense of stability has been discussed by Siddiqui^47^. Along with the pH 6.2 ink, lower reduction percentage was found for pH 7.2 (Fig. 2), however, for the former activity units were significantly higher. Additionally, at pH 6.2, a flat activity profile has been observed over a wide range of ionic strength^30^, and once again ensuring less influence on stability parameters. Considering the above discussion, pH 6.2 was designated as the optimum for next inkjetting steps.

Ionic strength (*I*) and valence of the salt used in enzyme solution are important factors for activity retention. Potassium salt has been found to be most permeable to the cell-walls for evident lytic action with an optimum value of *I* between 0.025 M to 0.12 M^27,30^. Accordingly, in our experiments, six lysozyme inks of varied potassium salt amount (*I* = 0.02–0.12 M) with optimized protein concentration and pH were jetted.

**Figure 3:**
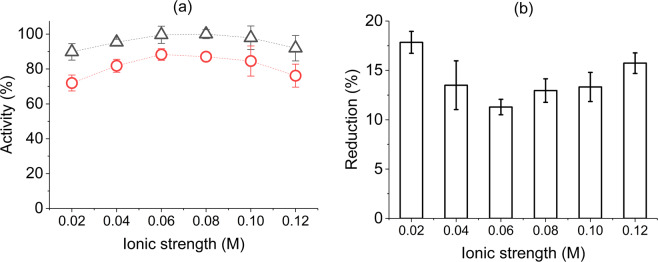
Effect of ionic strength on the activity of inkjetted lysozyme, (**a**) relative activity before (∆) and after (O) jetting when expressed as percentages of the highest value observed, (**b**) activity reduction after jetting process. Error bars represent standard deviation.

Relative activity of about 85% was found for inks of ionic strength 0.04–0.1 M with a trend to decrease gradually towards both extremes (0.02 and 0.12 M) as depicted in Fig. 3. Electrostatic forces regulating initial binding between lysozyme and the MLC of oppositely charged surface might be a reason for such activity trend. This might have resulted from an initial increase of reaction rate with an increment of *I* and then a gradual decrease due to affected charge distribution and varied amount of net charge on lysozyme surface^48^, along with a probability of shift in the pH-activity profile^28^. Davies *et al*.^30^ found similar behaviour to these prepared inks for lysozyme in buffer solution with pH 6.2 over wide range of *I*. Additionally, towards extreme ends of the used salt concentrations (0.02 M > *I* > 0.12 M) lysozyme has been observed to possess limited activity^49^, which indicates that inherent nature of lytic activity was not compromised on our formulated inks with varied *I*.

The activity trend after jetting was similar to that of non-jetted inks, but with reduced activity percentage (Fig. 3), particularly towards extreme *I* values (ca. 72% activity at *I* = 0.02 M). While activity might have been affected by restricted substrate binding at varied *I*, the same might have also caused a non-specific structural change to the protein conformation^49^. As discussed by Davies *et al*.^30^ and Yang and Honig^44^, in the absence of salt no activity would be expected for lysozyme, and at higher *I* values, stabilizing charges for protein conformation might have been inadequate to withstand the jetting force. Reduction of surface charge on lysozyme molecule with increasing pH and *I* have also been reported in other studies through measurements of *ζ*-potential^48,50^. In our results, reduction of activity percentages were relatively lower for inks with *I = *0.04–0.1 M (Fig. 3); nevertheless, considering the least percentage and possibility to maintain flat activity range over several pH levels^31^, *I* = 0.06 M was designated as optimum for inkjetting of lysozyme ink. In general, it can be concluded that jetting did not affect the activity retention of lysozyme inks for a wide range of ionic strengths as an optimized pH value was used.

### Effects of ink rheology

Viscosity and surface tension of ink solution are the two most important rheological parameters for ink ejection, continuous drop formation, and spreading on inkjet printed base-material. Since enzyme-based inks are water-based, these parameters need to be optimized by adding a proper amount of viscosity modifier and surfactant. These added reagents need to be compatible with each other, with the protein structure of the enzyme, and with printhead components. Additionally, they need to be effective over a range of pH, temperature and storage conditions.

#### Biphasic effect of ink viscosity and amount of viscosity modifier

Glycerol and PEG-400 have been widely used as viscosity modifiers for inkjet printing of various protein molecules and to improve overall printing efficiency^6,19,25,40,51^. Lysozyme has been found to be compatible with both of these modifiers and to have excellent solubility even at high concentrations^52,53^. Accordingly, four inks made with each of the modifiers were jetted at allowable viscosity range (5–20 mPa.s) of the printhead used. All of these inks had optimized protein concentration and pH-*I* profile following results from previous sections. Newtonian behaviour over a range of shear rates (max. 10000 s^−1^) was ensured for all our prepared inks. This helped to address viscosity fluctuation inside printhead caused by the actuation mechanism related to shear stress^2^.

**Figure 4:**
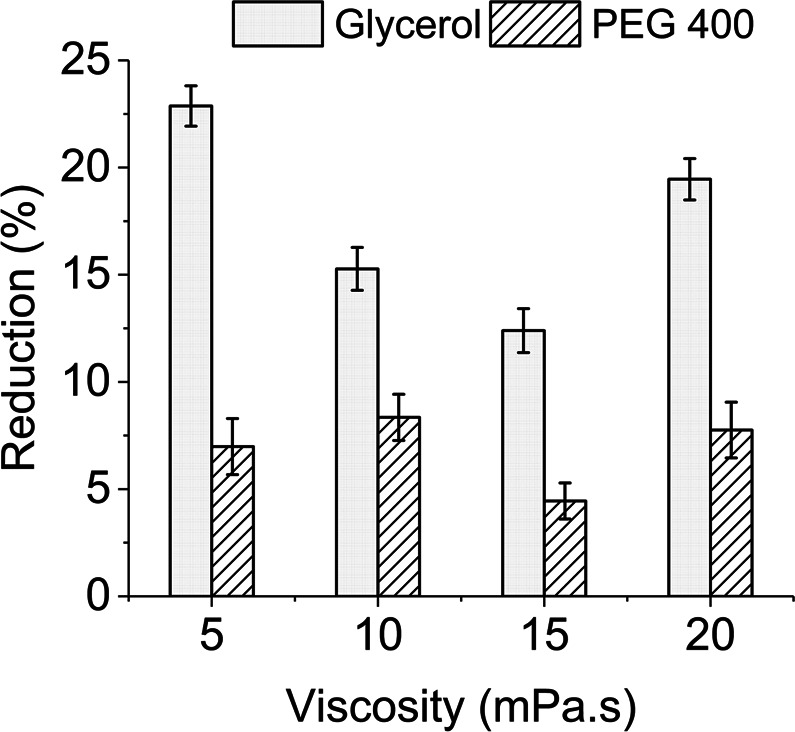
Activity reduction of inkjetted lysozyme against ink viscosities for two modifiers. Error bars represent standard deviation.

Inks produced with glycerol showed activity reduction of less than 15% at viscosity levels 10–15 mPa.s, in contrast to the two extreme viscosities, which had about 20% reduction (Fig. 4). PEG-400 containing inks had activity reduction of between 5–10% for all viscosity ranges (Fig. 4). Viscosity levels of the inks were increased by the addition of large polymers i.e. 30–70% v/v glycerol and 20–60% v/v PEG-400. Such addition might have limited redistribution ability of the polymers and caused self-association among enzymes by replacing the surrounding water molecules^54^. As indicated by Knubovets *et al*.^52^, the initial addition of these polymers could be helpful to stabilize the protein structure against the jetting force. However, adding more amounts of such large polymers may result in limited diffusion rate and thus less protection to the protein structure^55^ and thus, showing a biphasic effect. A similar tendency of activity reduction with an increased amount of glycerol have been found by Arrabito *et al*.^19^ for piezoelectric printing of GOx.

PEG-400 containing inks had better activity retention percentage (Fig. 4) but with a lower specific activity (max. 3,500 units/mL) when compared to glycerol-based inks (max. 4,700 units/mL). Reason for such improved retention might have been protection offered by PEG-400 to lysozyme protein structure^56^, however, macromolecular overcrowding caused by this large polymer might have caused lower specific activity^54^. PEG-400 has higher possibility to cause hindrance on enzyme diffusion rate by overcrowding, as its molar mass is almost four times higher than that of glycerol. Di Risio and Yan^25^ have reported a similar reduction in HRP activity by comparing PEG-200 to PEG-20,000.

These findings indicate that glycerol-based ink with a viscosity of 15 mPa.s (Fig. 4) is an optimum solution for inkjetting of lysozyme and accordingly was followed on later experimental steps. In addition, to modifying viscosity, glycerol can also work as a humectant to avoid unwanted evaporation and protein concentration variation on jetted inks.

#### Surface tension and ink solution stability

Triton-X has been found to be the most compatible non-ionic surfactant for lysozyme^14^. However, the concentration of the surfactant can be a concern in regulating enzyme activity and at the same time printing efficiency^7^. Hence, to study the effects of Triton-X concentration (0.002–0.1% w/w) and thereby surface tension on lysozyme activity retention, six inks were jetted with optimized parameters from previous steps.

**Figure 5:**
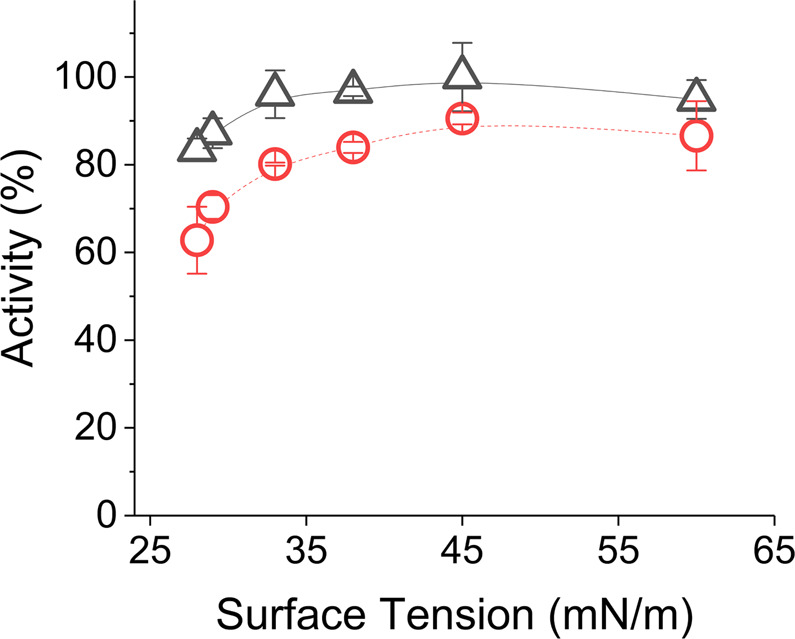
Relative activity of lysozyme ink at various surface tensions before (∆) and after (O) inkjetting process and expressed as percentages of the highest value observed. Error bars represent standard deviation.

Maximum activity was found for ink with Triton-X concentration of 0.007% (w/w) and surface tension of 45 mN/m (Fig. 5). After the initial increase, activity started to reduce gradually with a sharp fall near critical micelle concentration (*cmc*) at 29 mN/m. At the low surfactant concentration, the observed increase in activity could have been caused by phosphorylation related diffusion in enzyme membrane as explained by Griffin^57^ and Kagawa^58^. Contrary to this, increased surfactant amount might have caused additional solubilisation of protein membrane and activity reduction^14^. After jetting, the activity trend was similar, however, the activity retention percentage was reduced significantly for all surface tension levels. This reduction became larger as the inks approached lower surface tension values. It is possible that with the decrease of surface tension, the ink system became less stable to withstand the jetting force causing deformation of protein conformation and reduced activity. Di Risio (2007) found a similar effect on activity reduction with increased concentration of Triton-X for the ink containing HRP. Nevertheless, such a tendency cannot be generalized for all non-ionic surfactant. Bernath and Vieth^26^ found an increase in lysozyme activity with increased concentration of Tween-20, another commercial non-ionic surfactant when used above *cmc* point. Reason for such behaviour has been suggested to be the formation of a micelle-enzyme complex with preferential orientation of most active sites towards substrates. However, free enzymes would be preferred in many applications than as immobilized as formed with Tween-20. To avoid such micelle complex formation with enzymes, a surfactant concentration of just below *cmc* should be used for ink formulation. On the other hand, a well amount of surfactant usage is also necessary to ensure proper drop formation in DOD inkjet system^15^. Considering these findings, the surface tension value of 35 mN/m resulting about 85% activity retention can be considered as most effective for lysozyme ink.

### Ink storage

It is preferable to store enzymes at low temperature (4–8 °C) when used in solution form in order to ensure stable activity over a period^59^. However, it is desirable for ink solutions to be stored at room temperature to maintain a constant flow through the ink channel. Additionally, enzymes are expected to be used for several days after ink preparation^15^, during which they might undergo some structural changes and activity alteration due to storage time-temperature conditions. Solvents used to prepare ink solution, other than water, may also influence activity during storage due to the incubation effect^60^. To learn about these effects on glycerol-based lysozyme ink, three solutions of protein concentration 12, 25, and 50 µg/mL (linear range concentrations) were stored and jetted at room temperature for three consecutive days. Other ink parameters for these three set of inks were followed according to the results from previous steps.

**Figure 6:**
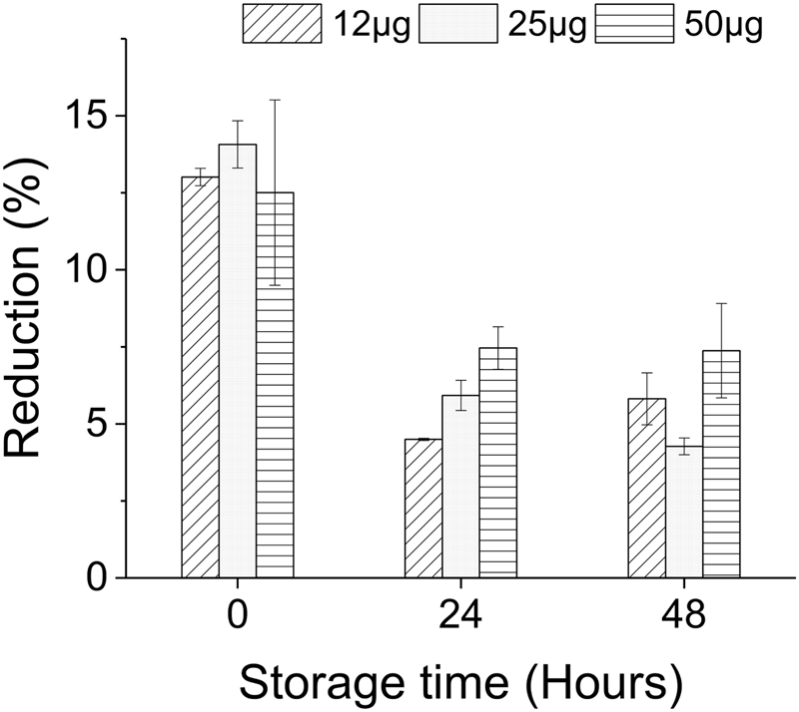
Activity reduction of lysozyme ink of three different protein concentrations when jetted after several hours of incubation at room temperature. Error bars represent standard deviation.

Glycerol-based lysozyme ink showed increased activity after 24 hours of storing at room temperature condition and continued the same up to 48 hours. Similarly, activity reduction after the jetting process significantly improved for all three inks of varying protein concentrations (Fig. 6). Reduction percentage became almost half (ca. 8%) after 24 hours compared to the inks jetted immediately after preparation (ca. 15%). However, no further significant improvement was visible onwards. Such improvement might have resulted from the incubation effect of lysozyme in glycerol that can enable better protein folding and an increase in activity at room temperature conditions^61^. Along with incubation time and temperature, the amount of glycerol present in ink formulation can influence the activity profile as well. A water-glycerol mixture may provide better folding yield and highly order native-like conformation to lysozyme than water alone. As found by Rariy and Klibanov^55^, the presence of 20–60% glycerol in water can provide ca. 50% improvement in protein folding. Consequently, when used in the right proportion, protection of protein structure can be enhanced in the presence of glycerol for lysozyme^52^. Our findings were well accordance with these explanations and showed an improvement in activity retention when jetted after hours of incubation.

The growth of any known bacteria or fungus on our lab environment was not observed during storage and thus did not influence the found enzymatic activity. Therefore, it can be summarized from our results that it is possible to inkjet print lysozyme inks for 2–3 consecutive days under room temperature, even with improved activity for certain formulations e.g. glycerol containing ink.

### Printhead temperature

Temperatures well above room condition provide good control throughout the printing process and an even flow of ink solution through nozzles^62^. However, the enzymes may show enhanced activity at an elevated optimum temperature when incubated for a certain duration^29^. Therefore, to learn about such effects, we jetted three glycerol-based lysozyme inks of varied concentration at printhead temperatures of 25, 40, 50, 60 °C after incubating at room condition for 12 hours. Other parameters were set to the optimum conditions as observed in our previous results.

**Figure 7:**
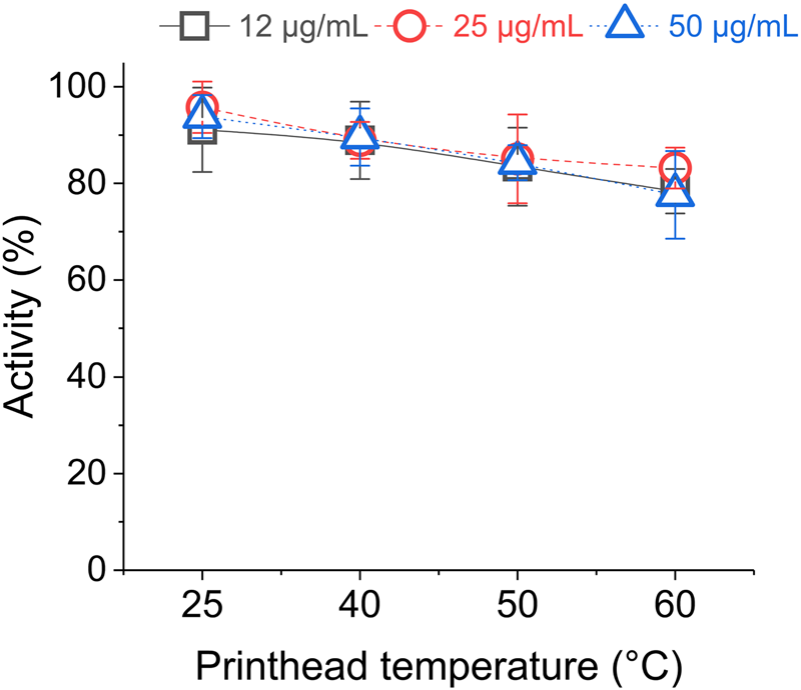
Relative activity of lysozyme inks with three different protein concentrations when inkjetted at several printhead temperatures and expressed as percentages of non-jetted control inks (25 °C). Error bars represent standard deviation.

An increasing trend of activity reduction percentage was observed with an increase of printhead temperature and became maximum of about 20% at 60 °C (Fig. 7). There might be several reasons for such a trend caused by elevated temperatures e.g. protein denaturation, increased glycerol content, and decreased viscosity. Glycerol, which may protect lysozyme against thermal denaturation^63^, can depress activity due to the increased amount by evaporation of water as discussed for Fig. 4. At the same time, transitory viscosity inside printhead may also influence activity by improper protein folding. However, the duration necessary for such unwanted folding to occur is more critical and might not have been long enough for our jetted inks to draw an obvious conclusion about this phenomenon. Similarly, literature suggested lysozyme denaturation above 70 °C when incubated for ca. 30 minutes^52^ were unlikely to happen for our jetted enzymes due to low jetting duration (3 minutes).

The effect of optimal temperature on lysozyme activity was not visible for our jetted inks (Fig. 7), which otherwise could be seen near 50 °C in a buffer solution as suggested in the literature^64^. Visibility of such temperature effect is more dependent on the duration of exposure which is usually around thirty minutes^65^. However, in our jetted samples such exposure was about three minutes leaving less possibility for optimal temperature effect on activity. Inks of different protein concentrations showed no significant variation of activity reduction and thus indicating the similar jetting ability of the linear range concentrations (12–50 µg/mL). To summarize, our findings indicate that it is possible to inkjet a well-optimized lysozyme ink over a range of printhead temperatures (25–50 °C) for various protein concentrations without significant loss of activity.

## Conclusion

In this paper, we have presented a comprehensive study on piezoelectric inkjet printing of lysozyme. Several effects of ink, printhead, and jetting operation related parameters have been discussed. Inks with low protein concentration were susceptible to shear stress inside printhead and resulted in comparatively low activity retention than higher protein concentrations. Activity retention of lysozyme was found to be greater for the piezoelectric system compared to its thermal counterpart.

The inkjetted enzyme showed an optimum pH and ionic strength level with comparatively better stability of protein structure against jetting force. Glycerol-water mixture of certain proportion provided efficient viscosity modification and activity retention upon incubation. Ink surface tension right above the critical micelle concentration of surfactant was suggested to ensure efficient printing with acceptable activity reduction. Optimum temperature effect was not observed for a heated printhead, rather inkjetting at near room temperature showed better activity retention. Overall findings of this paper indicate the parameters responsible for activity retention of inkjetted lysozyme and would be useful to follow for printing of other enzymes.
