# Author Correction: Effects of ink characteristics and piezo-electric inkjetting parameters on lysozyme activity

**DOI:** 10.1038/s41598-022-25674-5

**Published:** 2022-12-16

**Authors:** Tuser T. Biswas, Junchun Yu, Vincent A. Nierstrasz

**Affiliations:** Textile Materials Technology, Department of Textile Technology, Faculty of Textiles, Engineering and Business, University of Borås, Borås, Sweden

Correction to: *Scientific Reports* 10.1038/s41598-019-54723-9, published online 03 December 2019

The original version of this Article contains an error in the Results and Discussion section, under the subheading ‘Ink printability’,

“Velocity, density, characteristics length, and surface tension are denoted by *η, ρ, r* and *γ* respectively.”

should read:

“Velocity, viscosity, density, characteristics length, and surface tension are denoted by *ν, η, ρ, r* and *γ* respectively.”

